# Author Correction: Node-based generalized friendship paradox fails

**DOI:** 10.1038/s41598-023-34980-5

**Published:** 2023-05-17

**Authors:** Anna Evtushenko, Jon Kleinberg

**Affiliations:** 1 Department of Information Science, Cornell University, Ithaca, NY USA; 2 Department of Computer Science, Cornell University, Ithaca, NY USA

Correction to: *Scientific Reports* 10.1038/s41598-023-29268-7, published online 06 February 2023

The original version of the Article contained an error in Figure 4, where delta for nodes with degree 1 and 3 was incorrect. This change does not affect the conclusions of the Article.

The original Figure 4 and accompanying legend appear below.

**Figure 4:**
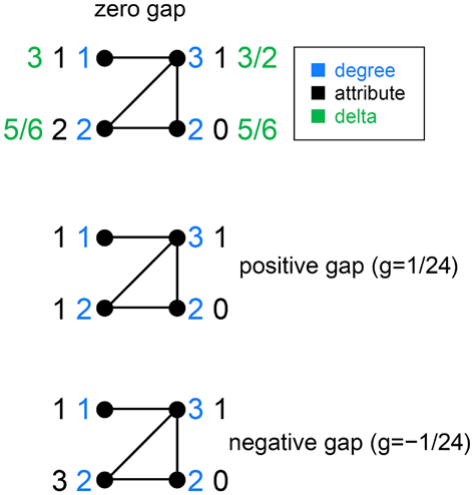
An anti-SGFP graph such as this may produce differently-signed gaps for *r*_*d*,*a*_ = 0, depending on the attribute sample.

The original article has been corrected.

