# Supplementary material for: Node-based generalized friendship paradox fails
Source: Sci Rep. 2023 Feb 6;13:2074. doi: 10.1038/s41598-023-29268-7 (PMC9902489; doi:10.1038/s41598-023-29268-7)
Supplement: Supplementary file 1 — Supplementary Information. [file 41598_2023_29268_MOESM1_ESM.pdf]

Node-based Generalized Friendship Paradox fails

Anna Evtushenko,[1*] Jon Kleinberg[1,2]

[1]Department of Information Science, Cornell University, Ithaca NY, USA
[2]Department of Computer Science, Cornell University, Ithaca NY, USA

*anna@infosci.cornell.edu

Supplementary Information

Contents

1 If all attributes or all degrees are the same, the gap is 0 **2**

1.1 All attributes are the same 2

1.2 All degrees are the same 2

2 Altering the attribute sample **3**

2.1 Adding a constant to each attribute doesn't change the gap 3

2.2 Scaling an attribute sample vector to length 1 doesn't change the degree-attribute correlation or the gap sign 3

3 $r_{d,a} = 1$ can't fail SGFP **4**

4 If all δ_i are equal in a connected graph, it's a regular graph **5**

1 If all attributes or all degrees are the same, the gap is 0

In the two cases below the degree-attribute correlation $r_{d,a}$ is undefined, so we can't use it to reason about the sign of the gap. So we look at the gap in each of these cases separately. For this section only our domain is connected graphs *including* regular graphs. Once we deal with regular graphs here, we don't consider them again.

1.1 All attributes are the same

If all attributes are the same and equal to x, each node i is averaging over the same values to get their second-order attribute s_i, so

$$g(\forall a_i = x) = \frac{1}{n}\sum_{i=1}^{n}\left(\left(\frac{1}{d_i}\sum_{j\in N(i)} a_j\right) - a_i\right) =$$

$$\frac{1}{n}\sum_{i=1}^{n}\left(\frac{1}{d_i}d_i x - x\right) = \frac{1}{n}\sum_{i=1}^{n}(x-x) = 0$$

1.2 All degrees are the same

Recall the definition of the gap in terms of δ_i from the main text:

$$g = \frac{1}{n}\sum_{j=1}^{n}\delta_j a_j - \frac{1}{n}\sum_{j=1}^{n} a_j$$

Note that in the case of all degrees being equal to y, $\delta_j = 1$ for each j, because there are y elements in the sum in δ_j (the number of elements is the number of j's friends) and each sum term is equal to $\frac{1}{y}$ (1 over the degree of each of j's friends):

$$\delta_j(\forall d_i = y) = \sum_{k\in N(j)}\frac{1}{d_k} = y\frac{1}{y} = 1$$

So the gap is equal to

$$g(\forall d_i = y) = \frac{1}{n}\sum_{j=1}^{n} a_j - \frac{1}{n}\sum_{j=1}^{n} a_j = 0$$

2 Altering the attribute sample

2.1 Adding a constant to each attribute doesn't change the gap

Call the SGFP gap for graph H and attribute sample a (expressed as a vector) $g_H(a)$. It is equal to

$$g_H(a) = \frac{1}{n}\sum_{i=1}^{n}\left(\frac{1}{d_i}\sum_{j\in N(i)} a_j\right) - \frac{1}{n}\sum_{i=1}^{n} a_i$$

$g_H(a)$ is unaffected if we add a constant vector c to a, effectively adding the same numeric constant to each node's attribute (call that constant C):

$$g_H(a+c) = \frac{1}{n}\sum_{i=1}^{n}\left(\frac{1}{d_i}\sum_{j\in N(i)} (a_j + C)\right) - \frac{1}{n}\sum_{i=1}^{n}(a_i + C) = \frac{1}{n}\sum_{i=1}^{n}\left(C + \frac{1}{d_i}\sum_{j\in N(i)} a_j\right)$$

$$-C - \frac{1}{n}\sum_{i=1}^{n} a_i = C + \frac{1}{n}\sum_{i=1}^{n}\left(\frac{1}{d_i}\sum_{j\in N(i)} a_j\right) - C - \frac{1}{n}\sum_{i=1}^{n} a_i =$$

$$\frac{1}{n}\sum_{i=1}^{n}\left(\frac{1}{d_i}\sum_{j\in N(i)} a_j\right) - \frac{1}{n}\sum_{i=1}^{n} a_i = g_H(a)$$

2.2 Scaling an attribute sample vector to length 1 doesn't change the degree-attribute correlation or the gap sign

As before, for a graph H and attribute sample a, we use $g_H(a)$ to denote the gap

$$\frac{1}{n}\sum_{i=1}^{n}\left(\frac{1}{d_i}\sum_{j\in N(i)} a_j\right) - \frac{1}{n}\sum_{i=1}^{n} a_i$$

Now, for a constant $\lambda > 0$, let λa be the attribute sample in which all values are scaled by λ: that is, the attribute value for node i is changed from a_i to λa_i.

We know that the correlation of two vectors is unchanged if we multiply either of them by positive scalar values. We also observe that the gap for λa is

$$g_H(\lambda a) \;=\; \frac{1}{n}\sum_{i=1}^{n}\left(\frac{1}{d_i}\sum_{j\in N(i)} \lambda a_j\right) - \frac{1}{n}\sum_{i=1}^{n} \lambda a_i$$

$$
\begin{aligned}
&= \frac{1}{n}\sum_{i=1}^{n}\left(\frac{\lambda}{d_i}\sum_{j\in N(i)} a_j\right) - \frac{\lambda}{n}\sum_{i=1}^{n} a_i \\
&= \frac{\lambda}{n}\sum_{i=1}^{n}\left(\frac{1}{d_i}\sum_{j\in N(i)} a_j\right) - \frac{\lambda}{n}\sum_{i=1}^{n} a_i \\
&= \lambda\left(\frac{1}{n}\sum_{i=1}^{n}\left(\frac{1}{d_i}\sum_{j\in N(i)} a_j\right) - \frac{1}{n}\sum_{i=1}^{n} a_i\right) \\
&= \lambda g_H(a).
\end{aligned}
$$

It follows that for any $\lambda > 0$, the attribute samples a and λa have the same correlation with the degree sequence, and also the same sign of their gaps $g_H(a)$ and $g_H(\lambda a)$.

Now, suppose we choose

$$\lambda = \frac{1}{\sqrt{\sum_{i=1}^{n} a_i^2}}.$$

Then λa has the property that the sum of its squared coordinates is equal to 1; that is, if $(\lambda a)_i$ denotes the ith coordinate λa, then $\sum_{i=1}^{n}(\lambda a)_i^2 = 1$. Thus, starting with a whose correlation is some value r, setting λ to this value and defining λa gives us an attribute sequence that also has correlation r, that produces a gap of the same sign that a does, and whose squared coordinates sum to 1.

3 $r_{d,a} = 1$ can't fail SGFP

An attribute sample with $r_{d,a} = 1$ can't fail SGFP.

Recall the gap formula from equation 1 of the main text:

$$g = \frac{1}{n}\sum_{i=1}^{n}\left(\frac{1}{d_i}\sum_{j\in N(i)} a_j\right) - \frac{1}{n}\sum_{i=1}^{n} a_i \tag{1}$$

$r_{d,a} = 1$ means that for each i, $a_i = \alpha + \beta d_i$ where $\beta > 0$. Plugging this into the gap

formula we get:

$$g(r_{d,a}=1)=\frac{1}{n}\left(\sum_{i=1}^{n}\frac{1}{d_i}\sum_{j\in N(i)}(\alpha+\beta d_j)\right)-\frac{1}{n}\sum_{i=1}^{n}(\alpha+\beta d_i)=$$

$$\frac{1}{n}\left(\sum_{i=1}^{n}\frac{1}{d_i}\sum_{j\in N(i)}(\alpha+\beta d_j)-\sum_{i=1}^{n}(\alpha+\beta d_i)\right)=$$

$$\frac{1}{n}\left(\sum_{i=1}^{n}\frac{1}{d_i}\sum_{j\in N(i)}\alpha+\sum_{i=1}^{n}\frac{1}{d_i}\sum_{j\in N(i)}(\beta d_j)-\sum_{i=1}^{n}\alpha-\sum_{i=1}^{n}(\beta d_i)\right)=$$

$$\frac{1}{n}\left(\sum_{i=1}^{n}\frac{1}{d_i}\alpha d_i+\beta\sum_{i=1}^{n}\frac{1}{d_i}\sum_{j\in N(i)}d_j-\sum_{i=1}^{n}\alpha-\beta\sum_{i=1}^{n}d_i\right)=$$

$$\frac{1}{n}\left(\alpha n+\beta\sum_{i=1}^{n}\frac{1}{d_i}\sum_{j\in N(i)}d_j-\alpha n-\beta\sum_{i=1}^{n}d_i\right)$$

And,

$$g(r_{d,a}=1)=\frac{\beta}{n}\left(\sum_{i=1}^{n}\frac{1}{d_i}\sum_{j\in N(i)}d_j-\sum_{i=1}^{n}d_i\right) \tag{2}$$

Recall that the Singular Friendship Paradox (SFP) is a special case of SGFP with degrees acting as attributes. The gap formula for SFP, adapting equation 1 above, is:

$$g_{SFP}=\frac{1}{n}\left(\sum_{i=1}^{n}\frac{1}{d_i}\sum_{j\in N(i)}d_j-\sum_{i=1}^{n}d_i\right) \tag{3}$$

Now compare that to $g(r_{d,a}=1)$ in equation 2. $g(r_{d,a}=1)=\beta g_{SFP}$ and, since β is positive, $g(r_{d,a}=1)$ has the same sign as g_{SFP}. That means that if SFP stands (and it does for all non-regular connected graphs, our domain here), so does SGFP in this specific case of $r_{d,a}=1$.

4 If all δ_i are equal in a connected graph, it's a regular graph

Given a connected graph $G=(V,E)$ on n nodes, and given a node j of G, we define $N(j)$ to be the set of neighbors of j; we define $d_j=|N(j)|$ to be the degree of j; and we define $\delta_j=\sum_{k\in N(j)}\frac{1}{d_k}$. In a non-trivial case of $|V|>1$, the set of neighbors of j is non-empty for all j and d_k is positive for all k, so we can expect δ_j to be defined and positive.

Claim: If G is connected and $\delta_i = \delta_j$ for all nodes i, j, then $d_i = d_j$ for all nodes i, j.

To prove this claim, we first observe that

$$\sum_{j \in V} \delta_j = \sum_{j \in V} \sum_{k \in N(j)} \frac{1}{d_k}$$

contains the term $\frac{1}{d_k}$ exactly d_k times (once for each of the d_k neighbors of k), and therefore

$$\sum_{j \in V} \delta_j = n.$$

It follows that if $\delta_i = \delta_j$ for all nodes i, j, then $\delta_i = 1$ for all nodes i.

Now suppose G contains two nodes whose degrees are not the same. Let a be a node of minimum degree and b be a node of maximum degree; so we have $d_a < d_b$. Since G is connected, there is a path P in G with one end equal to a and the other end equal to b. As we traverse P from a to b, there is a first node j on P for which $d_j \neq d_a$. Node j cannot be the first node on P, and the node i immediately preceding j on P has $d_i = d_a$, the minimum degree in G.

Now observe that $\delta_i = \sum_{k \in N(i)} \frac{1}{d_k}$ is a sum of d_i terms; each of these terms is at most $\frac{1}{d_i}$ since i has the minimum degree in G. Moreover, at least one of these terms is $\frac{1}{d_j} < \frac{1}{d_i}$. Therefore, since δ_i is a sum of d_i terms, each at most $\frac{1}{d_i}$ and one of the terms strictly less than $\frac{1}{d_i}$, we have $\delta_i < 1$.

Since $\delta_i \neq 1$, we conclude that G does not have the property that $\delta_i = \delta_j$ for all nodes i, j. It follows that the only connected graphs G with this property have the same degree at every node.

For graphs that are not connected, we can apply this argument to each connected component separately, and conclude that all the node degrees in each component are the same.
